# Genome and Transcriptome Analysis to Elucidate the Biocontrol Mechanism of *Bacillus amyloliquefaciens* XJ5 against *Alternaria solani*

**DOI:** 10.3390/microorganisms11082055

**Published:** 2023-08-10

**Authors:** Fan Mu, Xu Chen, Zhenxin Fu, Xue Wang, Jiexin Guo, Xiaojun Zhao, Baojun Zhang

**Affiliations:** Shanxi Key Laboratory of Integrated Pest Management in Agriculture, College of Plant Protection, Shanxi Agricultural University, Jinzhong 030801, China; fanmu1993@126.com (F.M.); s20212327@stu.sxau.edu.cn (Z.F.);

**Keywords:** *Bacillus amyloliquefaciens*, *Alternaria solani*, biocontrol, antifungal protein, secondary metabolites, chitin

## Abstract

Early blight, caused by *Alternaria solani*, is an important disease affecting tomatoes. Biological control offers an environmentally friendly approach to controlling pathogens. Herein, we identified a *B. amyloliquefaciens* strain XJ5 and investigated its biocontrol mechanism against *A. solani*. *A. solani* growth was significantly inhibited by XJ5, with the inhibition rate of cell-free culture supernatants reaching 82.3%. Furthermore, XJ5 crude protein extracts inhibited conidia germination and altered the mycelial morphology of *A. solani*. To uncover the potential biocontrol mechanism of XJ5, we analyzed its genome sequence and transcriptome. The genome of XJ5 comprised a 4.16 Mb circular chromosome and two circular plasmids. A total of 13 biosynthetic gene clusters and 127 genes encoding hydrolases were identified, suggestive of the ability of XJ5 to secrete antagonistic secondary metabolites and hydrolases. Transcript analysis revealed 174 differentially expressed genes on exposing *A. solani* to XJ5 crude protein extracts. The expression of genes related to chitin and mannose synthesis was downregulated, indicating that XJ5 metabolites may impact chitin and mannose synthesis in *A. solani*. Overall, these findings enhance our understanding of the interactions between *B. amyloliquefaciens* and phytopathogens and pave the way for the agricultural application of this promising biocontrol agent.

## 1. Introduction

Early blight of tomato is one of the most devastating diseases caused by the necrotrophic pathogen *Alternaria solani*, which is distributed worldwide [1]. The pathogen can infect leaves, stems, petioles, twigs, and fruits under favorable conditions, causing approximately 50–86% loss in fruit yield [2]. Fungicide treatments are the most efficient approach for controlling early blight; however, the frequent and incorrect application of agrochemicals can lead to the emergence of resistant pathogens and adversely impact the environment [3,4,5]. Biological control of early blight has received increased attention, and various microorganisms and their metabolites with antifungal activity against *A. solani* have been reported [2,6].

*Bacillus* spp. have attracted much attention due to their characteristics of inhibiting the growth of pathogens and their potential as biocontrol agents [7,8]. These species are capable of producing a broad range of antimicrobial substances via nonribosomal peptide synthetase (NRPs) and ribosomal synthetase to suppress the growth of phytopathogens [7,9]. The antimicrobial compounds synthesized via NRPs include lipopeptides, polyketides, and other antibiotics. Lipopeptides typically comprise four families: surfactin, iturin, fengycin, and kurstakins [10]. These are known to inhibit the growth of fungi and bacteria or induce systemic resistance in host plants [11,12,13,14]. Polyketides and other antibiotics (bacilysin and bacteriocin) exhibit strong bactericidal activity against fungi, bacteria, and algae [15,16,17,18,19]. Antimicrobial substances synthesized through the ribosomal pathway mainly include bacteriocins (subtilin, sublancin 168, and subtilosin), hydrolytic enzymes (e.g., extracellular amylase, chitinase, and glucanases), and other unidentified antimicrobial proteins [20,21,22]. The chitinase produced by *Bacillus* spp. is effective in inhibiting pathogenic fungi [23,24]. Moreover, *Bacillus* spp. can produce various types of volatile organic compounds to promote plant growth and hamper fungal growth [25,26].

Genomics and transcriptomics have provided new insights into the genetic basis of *Bacillus* spp. and their interaction with plant pathogens. For instance, *B. subtilis* TY-1 reportedly significantly inhibits *Ralstonia solanacearum*, and a large number of gene clusters involved in antibacterial metabolite synthesis are present in the genome of TY-1, demonstrating the potential of TY-1 as a biocontrol agent for tobacco bacterial wilt [27]. The genome of *B. amyloliquefaciens* AS 43.3 provides a foundation for understanding the mechanism of this strain and its biocontrol interaction with Fusarium head blight [28]. The genome analysis of *B. cereus* B25 revealed the involvement of numerous chitinases, glycoside enzymes, and antibiotics against the maize pathogenic fungus *Fusarium verticillioides* [29]. The antifungal activity of *B. subtilis* SG6 may be related to the synergistic production of chitinase, fengycins, and surfactins [30]. These findings indicate that gene clusters associated with secondary metabolite synthesis and hydrolytic enzymes are related to potential biocontrol activity against phytopathogens. Meanwhile, the mechanisms of *Bacillus* spp. as a biocontrol agent have been explored through transcriptome analysis. For example, *B. amyloliquefaciens* JDF3 and *B. subtilis* RSS-1 can evidently inhibit the growth of *Phytophthora sojae* by suppressing ribosome activity [31]. Furthermore, *B. subtilis* induces disease resistance in wheat by upregulating the expression of a plant hormone signal pathway gene, namely *nonexpressor* of pathogenesis-related genes 1, to control wheat powdery mildew [32].

*B. amyloliquefaciens* is an important biocontrol bacterium that promotes the growth of plants and suppresses the growth of phytopathogens [33]. *B. amyloliquefaciens* harbors diverse functional genes related to biocontrol traits, ensuring its biocontrol effect [28,33,34]. However, there have been few reports on the inhibitory mechanism of *B. amyloliquefaciens*. In this study, a *B. amyloliquefaciens* strain XJ5 was identified and its biocontrol mechanism against the phytopathogen *A. solani* was studied. Crude protein extracts from XJ5 were observed to inhibit the hyphal growth and conidia germination of *A. solani*. The biocontrol mechanism of XJ5 was elucidated upon genome sequence and transcriptome analyses. Our data provide the basis for the inhibitory mechanism of *B. amyloliquefaciens* on plant pathogens and can facilitate the development of *B. amyloliquefaciens* into a biofungicide.

## 2. Materials and Methods

### 2.1. Strains and Culture Conditions

*B. amyloliquefaciens* XJ5 (GenBank accession MT000964) was isolated and cultured in beef peptone yeast (BPY) media (5 g/L beef extract, 10 g/L peptone, 5 g/L yeast extract, 5 g/L sodium chloride, and 5 g/L dextrose, pH 7.0) at 25 °C and maintained at −80 °C in 20% glycerol. *A. solani* was cultured in potato dextrose agar (PDA) media (glucose: 20 g, potato: 200 g and agar 10 g, 1 L ddH_2_O) at 25 °C, and maintained on PDA slants at 4 °C.

### 2.2. In Vitro Antifungal Activity Assays

Dual culture assays were performed to evaluate the in vitro antifungal activity of *B. amyloliquefaciens* against *A. solani. A. solani* was cultivated on PDA for 5 days at 25 °C; subsequently, fresh hyphae discs from *A. solani* colony margins, measuring 6 mm in diameter, were placed on a new PDA plate, and cell suspension of XJ5 was spread in a straight line on both sides of the disc [35]. A fresh disc from *A. solani* colony margins (diameter: 6 mm) was inoculated on PDA as the control. All PDA plates were inoculated at 25 °C for 5 days. There were at least four biological repeats for each treatment, and the assay was replicated twice. The inhibition rate (IR) was calculated according to a previously described method [35]: IR (%) = (colony diameter of control *A. solani* − colony diameter of *A. solani* treated with XJ5) / colony diameter of control *A. solani* × 100%.

### 2.3. Antifungal Activity Assessment of Crude Protein Extracts from XJ5

The protein component was harvested using a previously reported method with some modifications [36]. XJ5 cells were streaked onto BPY agar plates, and a single colony was inoculated into 100 mL BPY broth and cultured at 25 °C for 72 h with shaking at 200 rpm. This entire culture was then centrifuged at 4 °C, and 12,000 rpm for 10 min. Subsequently, it was filtered through a 0.22-µm filter to obtain cell-free culture supernatant. The supernatant was cooled on ice, followed by the addition of saturated ammonium sulfate solution to bring the ammonium sulfate concentration to 70% saturation. This mixture was then incubated at 4 °C for 2 days. The precipitation products were collected by centrifugation at 4 °C and 10,000 rpm for 5 min, dissolved in Tris-HCL buffer (10 mM, pH 7.4), and dialyzed using dialysis bags (Molecular Weight Cut-Off 8000–14,000, Solarbio, Beijing, China) at 4 °C for 12 h. Precipitates were freeze-dried, dissolved in distilled water, and filtered through a 0.22-µm filter to obtain crude protein extracts. Then, 50 µL spore suspensions of *A. solani* (2 × 10^5^) were inoculated on a PDA plate; using a sterilized punch, two wells (6 mm in diameter) were then created on the plate 2 cm from the dried spore suspension, crude protein extracts (100 μL) were pipetted into one of the wells, and ddH_2_O (100 μL) without crude protein extracts was pipetted into the other well as a control.

### 2.4. Light Microscopy and Scanning Electron Microscopy (SEM) of A. solani

*A. solani* conidia were observed under a light microscope 48 h after incubation with crude protein extracts from XJ5. *A. solani* hyphae disc (diameter: 6 mm) was inoculated into 100 mL potato dextrose broth, which contained crude protein extracts from XJ5 at a concentration of 1% and cultivated with constant shaking at 25 °C and 120 rpm for 6 h, 12 h, 24 h, and 48 h, respectively. Afterward, hyphae were collected and fixed with a solution containing 2.5% glutaraldehyde, followed by dehydration using progressively increasing concentrations of ethanol (30%, 50%, 70%, 80%, 90%, 95%, and 100%). The samples were dried with a critical point drier using the critical-point drying method and observed under a scanning electron microscope (JSM-6490LV, JEOL Ltd., Tokyo, Japan). *A. solani* hyphae grown on a PDA plate served as a control.

### 2.5. DNA Extraction, Genome Sequencing, Assembly, and Annotation

A sodium dodecyl sulfate (SDS) based method, as previously described, was used to extract genomic DNA from XJ5 cells [37]. The harvested DNA was detected by agarose gel electrophoresis and sequenced using the PacBio Sequel platform and Illumina NovaSeq PE150 (Novogene Bioinformatics Technology, Beijing, China). Glimmer 3.02 was used to predict coding sequences [38]. Tandem Repeats Finder, 4.07b, was utilized to analyze tandem repeat sequences [39]. tRNAscan-SE 2.0 was used to analyze transfer RNA (tRNA) genes [40]. RNAmmer and CMsearch were used to predict ribosome RNA (rRNA) genes and small nuclear RNA (snRNA), respectively [41,42]. Predicted genes were annotated using six databases (accessed on 20 October 2022): Gene Ontology (GO, http://www.geneontology.org/), Clusters of Orthologous Groups (COG, http://eggnog.embl.de/), Kyoto Encyclopedia of Genes and Genomes (KEGG, http://www.genome.jp/kegg/), Non-Redundant Protein Database (NR, ftp://ftp.ncbi.nlm.nih.gov/blast/db/), Pfam (http://pfam-legacy.xfam.org/), and Swiss-Prot (https://web.expasy.org/docs/swiss-prot_guideline.html). Carbohydrate-active enzyme annotation analysis was performed using the CAZy database. The secondary metabolism gene clusters were predicted using antiSMASH 4.0.2 [43]. The annotation and classification of protein-coding genes provided the basic information for the functional annotation of the strain genome.

### 2.6. Total RNA Extraction, RNA Sequencing, and Transcriptomic Analysis

*A. solani* hyphae treated with crude protein extracts from XJ5 for 6 h, 12 h, 24 h, and 48 h were collected. *A. solani* hyphae grown on PDA served as a control. Each treatment consisted of three biological replicates, and fifteen samples were collected. Hyphae were ground in liquid nitrogen with a mortar and pestled to a fine powder. Total RNA was extracted using a TRIzol RNA Extraction Kit (Takara Bio, Inc., Kusatsu, Japan) as recommended by the manufacturer. Nanodrop 2000 (Thermo Scientific™, Waltham, MA, USA) and agarose gel electrophoresis were used to check RNA concentrations. mRNA enrichment and library preparation were performed, and the libraries were sequenced on the Illumina NovaSeq 6000 platform (Majorbio Bio-Pharm Technology Corporation, Shanghai, China), with each library generating >6 Gb data. The raw data were deposited in the National Center for Biotechnology Information (NCBI) Sequence Read Archive (SRA) Database under the accession number PRJNA989022. The raw reads from 15 libraries were processed to remove adaptor sequences, unqualified reads with a high content of unknown base (N) reads (content > 10%), and those with a low-quality score (<10). HISAT2 (v2.1.0) was used to map the clean reads to the reference genome of *A. alternata* (accession number: GCF_001642055) [44], and bam files were then obtained. StringTie2 was utilized to assemble the bam files with default parameters [45,46]. Gene expression levels were extracted using the prepDE.py script provided by StringTie2. Differentially expressed genes (DEGs) were analyzed using the DESeq2 package in R with the following filtering criteria: adjusted *p*-value < 0.05 and |log2 (fold change) | > 1 [47]. DEGs were annotated in six different databases (NR, Swiss-Prot, Pfam, eggNOG, GO, and KEGG). GO functional annotation was performed using Blast2GO (version:5.2, https://www.blast2go.com/). KEGG annotation was conducted using KOBAS 3.0 [48]. GO and KEGG pathway enrichment analyses were performed using the clusterProfiler (v4.8.1) package in R with default parameters. Correlations between samples were calculated using Pearson’s correlation coefficient and principal component analysis (PCA).

### 2.7. Real-Time Quantitative Reverse Transcription PCR (qRT-PCR) Analysis

For qRT-PCR, *A. solani* cDNA was synthesized with an oligo d(T) primer using a PrimeScript™ RT Reagent Kit with gDNA Eraser (Perfect Real Time) (Takara Bio, Inc., Kusatsu, Japan) according to the manufacturer’s instructions. Beacon Designer v7.92 was used to design gene-specific primers for selected DEG sequences (Supplementary Table S1). qRT-PCR was performed on the CFX96 real-time PCR detection system (Bio-Rad, Hercules, CA, USA) with TB Green^®^ Premix Ex Taq™ II (Takara Bio, Inc., Kusatsu, Japan). The PCR reaction condition is as follows: pre-denaturation at 95 °C for 30 s, followed by 40 cycles of denaturation at 95 °C for 5 s, and extension at 60 °C for 30 s. *A. solani* actin gene (GenBank: MK388241.1) was used as an internal reference gene. The relative expression levels of each DEG were calculated using the 2^−ΔΔCT^ method [49], and qRT-PCR results were then compared with RNA-seq data.

## 3. Results

### 3.1. Morphological Observation and Antifungal Activity of B. amyloliquefaciens XJ5

**Figure 1:**
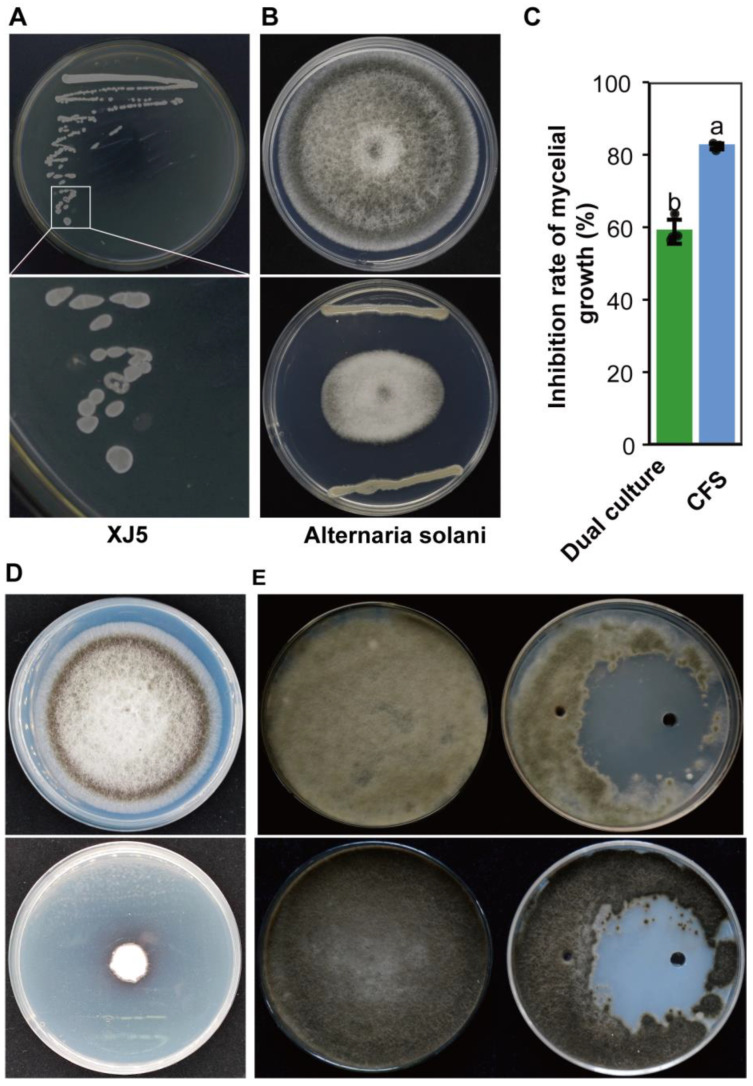
Morphological observation and antifungal activity of *B. amyloliquefaciens* XJ5. (**A**) Growth phenotype of XJ5 on BPY media. (**B**) Antifungal activity of XJ5 against *A. solani* as assessed by dual culture assay. (**C**) Inhibition rate of *A. solani* mycelial growth as assessed for both dual culture and CFS. Dual culture: the inhibition rate of *A. solani* dual culture with strain XJ5. CFS (cell-free culture supernatants): the inhibition rate of *A. solani* treated with the CFS of XJ5. Error bars represent the standard deviation from four sample means, and different letters on the top of each column indicate significant differences at the *p* < 0.05 level of confidence according to the *t* test. (**D**) Antifungal activity of CFS of XJ5. (**E**) Inhibitory activity of XJ5 crude protein extracts against *A. solani*. Colony morphology of *A. solani* after 3 days (top) and 5 days (bottom) of treatment with XJ5 crude protein extracts.

XJ5 was observed to grow as white, smooth-faced, nontransparent colonies after 24 h of cultivation on BPY agar plate (Figure 1A). The dual culture assays indicated that XJ5 inhibited the mycelial growth of *A. solani* on PDA, resulting in a clear inhibition zone (Figure 1B,C). Further, the cell-free culture supernatants of XJ5 were found to suppress the mycelial growth of *A. solani*, and *A. solani* colony growth diameters decreased by 82.3% (Figure 1C,D). To further investigate the antifungal constituents of XJ5, crude protein was extracted from the fermentation supernatant broth of XJ5, and its antifungal activity was analyzed. After 3 days of treatment with crude protein extracts from XJ5, *A. solani* growth was found to be inhibited, with an inhibition zone diameter of 48 mm (Figure 1E); a significant antifungal effect was observed even 5 days after treatment (Figure 1E). These results indicated that XJ5 inhibits the mycelial growth of *A. solani* by secreting antifungal proteins.

### 3.2. XJ5 Inhibits Conidia Germination and Disturbs the Hyphal Structure of A. solani

**Figure 2:**
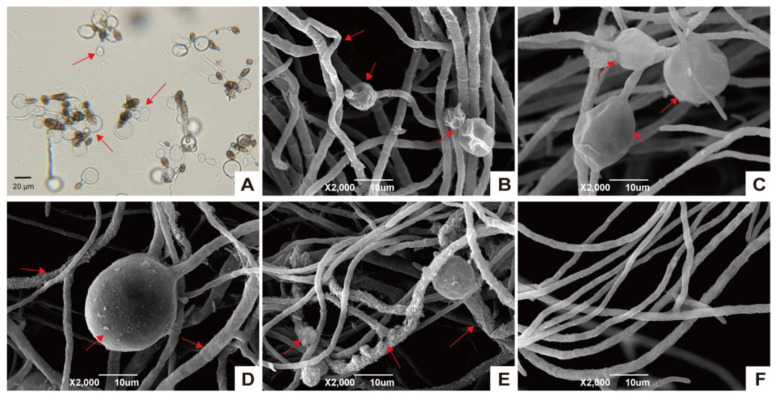
Microscopic morphology observations of *A. solani* treated with crude protein extracts from XJ5. (**A**) Conidia morphology of treating *A. solani* with crude protein extracts from XJ5 for 48 h; (**B**–**E**) SEM images showing hyphal morphology of *A. solani* treated with XJ5 crude protein extracts for 6 h, 12 h, 24 h, and 48 h, respectively. Red arrows indicate hyphal malformation. (**F**) The hyphae of *A. solani* grown on PDA plate observed by scanning electron microscopes were used as the negative control.

*A. solani* was treated with crude protein extracts from XJ5, and its morphology was observed by light microscopy and SEM. Microscopic observation revealed that the germination and germ tube elongation of *A. solani* conidia were inhibited on treatment with crude protein extracts from XJ5 (Figure 2A). The SEM observation showed that *A. solani* hyphae in the control group appeared plump and smooth and showed clear outlines (Figure 2F), while *A. solani* hyphae treated with crude protein extracts from XJ5 appeared swollen and enlarged (Figure 2B–E). After 6 h of treatment with crude protein extracts from XJ5, *A. solani* mycelium was swollen and showed spherical vesicles with surface wrinkles (Figure 2B); after 12 h of treatment, spherical vesicles were enlarged, and their abundance was higher (Figure 2C). Further, after 24 h of treatment, spherical vesicles were expanded and hyphal cell walls appeared rough and shriveled (Figure 2D), and after 48 h of treatment, damage and distortion of hyphae were apparent, and degradation of some mycelia was evident (Figure 2E). These results suggested that crude protein extracts from XJ5 target the cell wall of *A. solani* to exert their inhibitory effect against this tomato early blight fungus.

### 3.3. Genome Features of B. amyloliquefaciens XJ5

**Figure 3:**
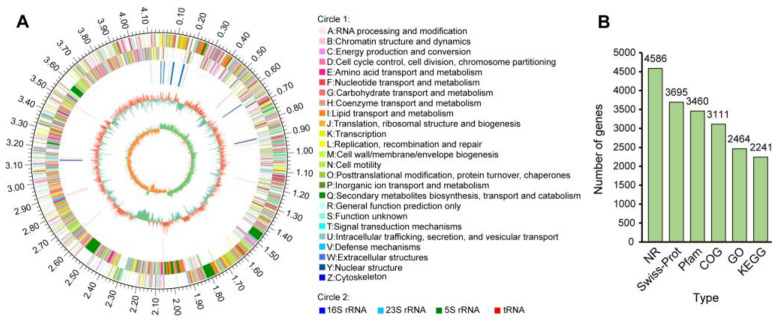
Genome data of *B. amyloliquefaciens* XJ5. (**A**) Graphical circular map of the genome. From the outside to the center: Circle 1, the size of the genome; Circle 2, gene CDS on the forward strand; Circle 3, gene CDS on the reverse strand; Circle 4, rRNA (blue: 16S rRNA, cyan: 23S rRNA, and green: 5S rRNA) and tRNA (red); Circle 5, GC content on the forward strand; Circle 6, GC content on the reverse strand; Circle 7, GC skew on the forward strand; and Circle 8, GC skew on the reverse strand. (**B**) Number of predicted genes found in several databases.

The *B. amyloliquefaciens* XJ5 genome was sequenced to elucidate its biocontrol mechanism. The genome features of XJ5 are summarized in Figure 3 and Supplementary Table S2. The genome of *B. amyloliquefaciens* XJ5 showed a circular chromosome (4,160,003 bp) and two plasmids (215,881 and 63,621 bp); the GC content was 46.09%, 37.28%, and 40.93%, respectively (GenBank: CP071970.1, CP071971.1, and CP071972.1, respectively). There was a total of 4199 predicted protein-coding genes in the chromosome; 310 in the first plasmid, and 77 in the second plasmid. The total length of protein-coding genes was 3,939,753 bp, accounting for 88.74% of the genome sequence. A total of 43 tandem repeat sequences, 88 tRNA-coding genes, and 27 rRNA genes were predicted in the chromosome sequence (Figure 3A). The number of genes annotated in NR, Swiss-Prot, Pfam, COG, GO, and KEGG databases was 4586, 3695, 3460, 3111, 2464, and 2241, respectively (Figure 3B).

### 3.4. Potential Functional Genes Involved in Biocontrol Traits

An analysis of the genome annotation of *B. amyloliquefaciens* XJ5 revealed several potential functional genes associated with biocontrol traits. XJ5 was found to contain numerous functional genes involved in the synthesis of hydrolytic enzymes and secondary metabolites. According to KEGG analysis, 1308 genes were annotated to metabolism pathways, with 240, 205, 160, and 117 genes enriched in carbohydrate metabolism, amino acid metabolism, metabolism of cofactors and vitamins, and energy metabolism, respectively (Supplementary Figure S1A). Within carbohydrate metabolism, dominant pathways included amino sugar and nucleotide sugar metabolism (KO00520), starch and sucrose metabolism (KO00500), and glycolysis/gluconeogenesis (KO00010). Forty-two genes were associated with KO00520, including a gene encoding chitosanase (EC: 3.2.1.132), which exhibits antifungal activity against fungi containing chitosan components in their cell walls. Furthermore, genes related to β-1,4-endoglucanase (*eglS*) and β-1,3-1,4-endoglucanase (*bglS*) were identified, which degrade fungal cell walls by hydrolyzing β-1,3-glucosidic bonds. A total of 127 genes encoding hydrolytic enzymes were annotated using the CAZy database (Supplementary Figure S1B), which were distributed across six CAZyme subfamilies, glycoside hydrolases (GHs, n = 42), glycosyl transferases (GTs, n = 39), carbohydrate esterases (CEs, n = 32), auxiliary activities (AAs, n = 9), polysaccharide lyases (PLs, n = 3), and carbohydrate-binding modules (CBMs, n = 2). Within glycoside hydrolases, forty-two genes were identified, including three annotated as chitinases, nine with potential cellulose-degrading ability, and one encoding chitosanase (*csn*). These findings demonstrated that the genome of *B. amyloliquefaciens* XJ5 has the potential to degrade cellulose, hemicellulose, chitin, pectin, and glucan.

Thirteen secondary metabolite biosynthetic gene clusters (BCGs) were found in the genome of XJ5 (Table 1), including five NRPs (locillonycin, surfactin, fengycin, bacillibactin, and a gene cluster that is not matched to a known NRPS), two polyketide synthetases (butirosin A/butirosin B), three trans-acyltransferase polyketide synthetases (macrolactin H, bacillaene, and difficidin), two terpenes, and one other BCGs (bacilysin). These BCGs reportedly exhibit high efficacy against bacteria, fungi, and viruses. The production of antimicrobial peptides through the nonribosomal synthesis pathway is an important mechanism employed by biocontrol bacteria to suppress phytopathogens. These findings indicated that the antagonistic activity of *B. amyloliquefaciens* XJ5 against *A. solani* may be related to the synthesis of these biocontrol agents. Furthermore, four functional unknown gene clusters (clusters 4, 8, 9, 12) were found, including two terpenes, one type III polyketide synthase (T3PKS), and one NRPS, indicating that the presence of additional gene clusters in XJ5 for the synthesis of potential novel antifungal substances. Consequently, XJ5 may have significant application potential in agriculture.

### 3.5. Transcriptomic Changes in A. solani Treated with B. amyloliquefaciens XJ5

**Figure 4:**
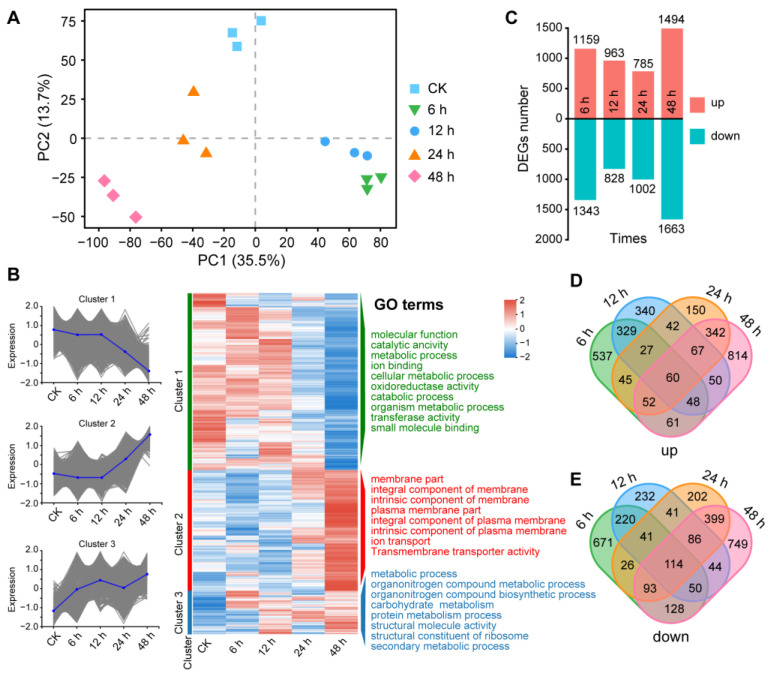
Differential expression genes of *A. solani* treated with crude protein extracts from XJ5. (**A**) Principal component analysis (PCA) showing the relatedness among the three biological replicates of RNA-seq samples. PCA was plotted by the first two axes (PC1 and PC2). Different colored shapes represent different samples. (**B**) GO analysis of DEGs in each sample. (**C**) Number of DEGs between different samples. The X-axis displays different comparison samples, and the Y-axis displays the number of genes. Red represents upregulated DEGs, and cyan represents downregulated DEGs. (**D**) Venn diagram showing the overlap of upregulated genes among different samples, numbers in the overlapping area indicate the number of genes that were co-upregulated between different samples. (**E**) Venn diagram shows the overlap of downregulated genes among different samples; numbers in the overlapping area indicate the number of genes that were co-downregulated between different samples.

Upon sequencing, each sample generated an average of 6.5 Gb raw reads; 745,252,900 clean reads were eventually obtained, with Q30 value > 96%. The genome mapping rates for reads from different samples ranged between 82.27% and 86.79% (Supplementary Table S3). PCA indicated a relatively high correlation between sequenced duplicate samples (Figure 4A). After 6 h, 12 h, 24 h, and 48 h of treatment with crude protein extracts from XJ5, 2502, 1791, 1787, and 3157 DEGs were identified, respectively, and they were grouped into three clusters (Figure 4B,C). Cluster 1 was mainly enriched in molecular function (GO:0003674), metabolic process (GO:0008152), ion binding (GO:0043167), and catalytic activity (GO:0003824), and the expression of the involved genes was generally downregulated (Figure 4B). Cluster 2 was mainly enriched in the membrane part (GO:0044425), an integral component of the membrane (GO:0016021), an integral component of the plasma membrane (GO:0005887), an intrinsic component of the plasma membrane (GO:0031226), and the expression of the involved genes was generally upregulated (Figure 4B). Cluster 3 was mainly enriched in the metabolic process (GO:0008152), carbohydrate metabolic process (GO:0005975), and secondary metabolic process (GO:0019748). Overall, 174 genes were differentially expressed in total, of which the expression of 60 genes was upregulated and that of 114 genes was downregulated (Figure 4D,E).

**Figure 5:**
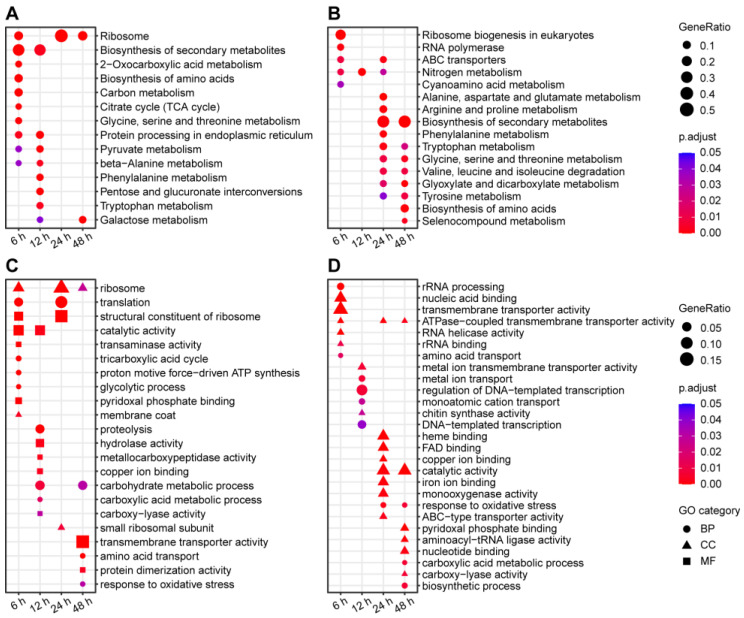
Enrichment analysis of differential expression genes. (**A**) GO analysis of upregulated genes. Bubble chart depicting the GO enrichment of upregulated genes after 6 h, 12 h, 24 h, and 48 h of treatment. The bubble color indicates the P value of the hypergeometric test for GO enrichment, and the bubble size indicates the ratio of the GO term relative to the background gene set. (**B**) GO analysis of downregulated genes. (**C**) KEGG pathway enrichment analyses of upregulated genes. (**D**) KEGG pathway enrichment analyses of downregulated genes.

**Figure 6:**
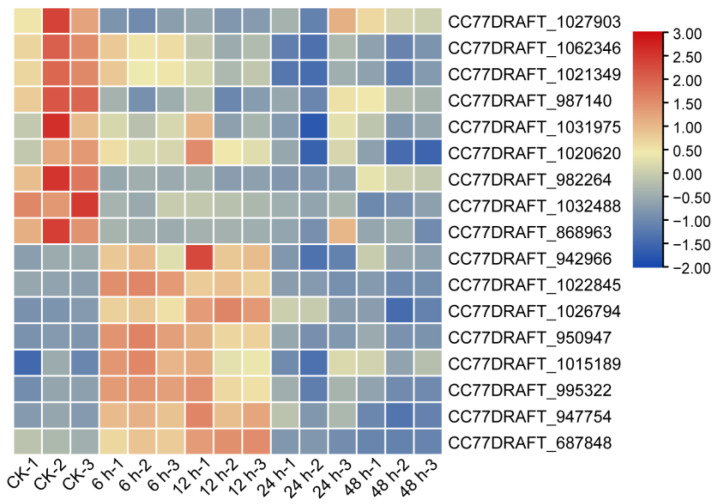
Expression pattern of chitin synthase, O-glycosylation, and N-glycosylation-related genes after 6 h, 12 h, 24 h, and 48 h of treatment. Heatmap was plotted using Z-score normalized transcript per million (TPM) values of the related genes by TBtools. Columns represent replicates of each sample and rows represent individual genes. Upregulated genes are in red, and downregulated genes are in blue.

**Figure 7:**
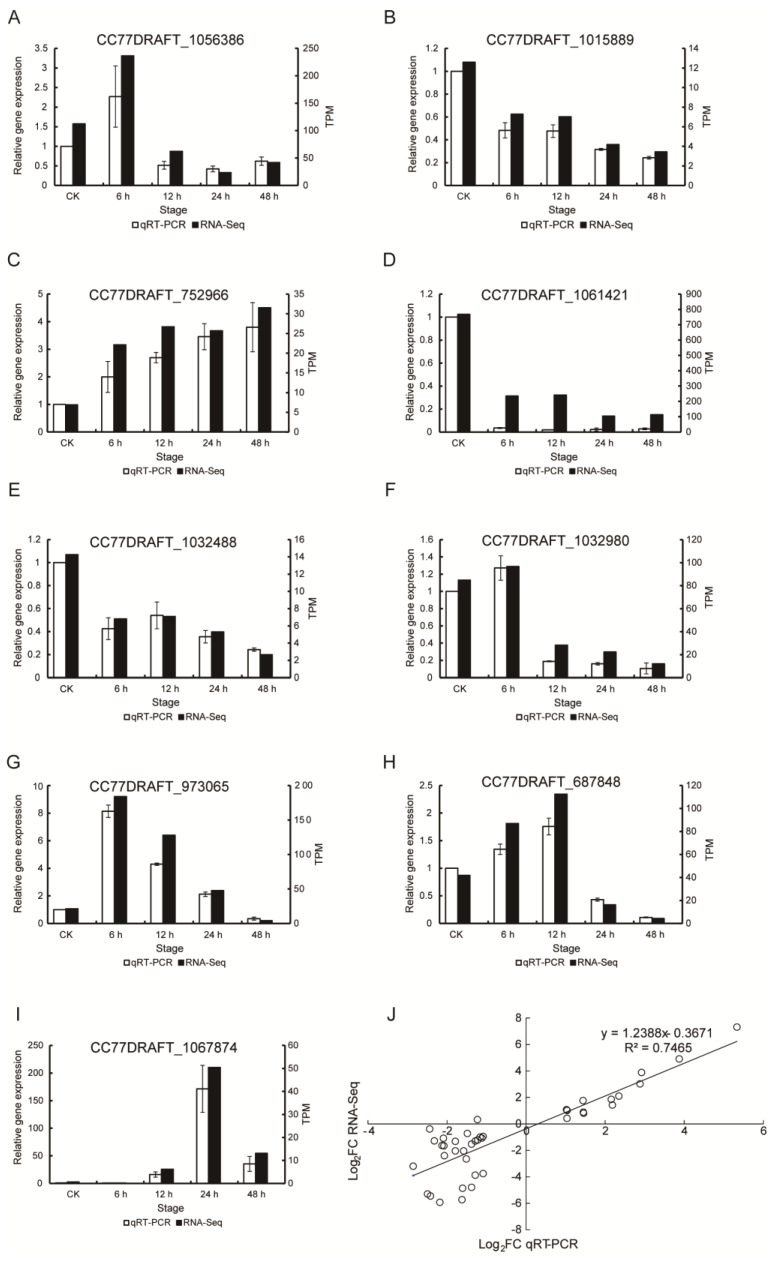
Validation of RNA-seq data by qRT-PCR. (**A**–**I**): Expression patterns of nine expressed genes in various databases. (**J**) Correlation dot plot between RNA-seq and qRT-PCR expression patterns.

After 6 h and 12 h of treatment with crude protein extracts from XJ5, a higher number of upregulated genes was identified, which were primarily enriched in terms such as ribosome and secondary metabolite synthesis (Figure 5A). KEGG pathway analysis indicated enrichment in ribosome pathways, structural constituents of ribosomes, catalytic activity, and others (Figure 5C). After 48 h of treatment, there were more downregulated genes, mainly enriched in pathways such as biosynthesis of amino acids and ATPase-coupled transmembrane transporter activity (Figure 5B,D). In addition, the expression of genes involved in cell wall synthesis, such as chitin synthase and mannose synthase-related genes, including CC77DRAFT_1027903, CC77DRAFT_1062346, CC77DRAFT_1021349, CC77DRAFT_987140, CC77DRAFT_1031975, CC77DRAFT_1020620, CC77DRAFT_1022845, and CC77DRAFT_868963, exhibited significant downregulation across all four stages of treatment, potentially impacting chitin and mannose synthesis in *A. solani* (Figure 6). The expression of genes related to glutathione S-transferases (GSTs), O-mannosylation, and the N glycan-processing pathway, including CC77DRAFT_1026794, CC77DRAFT_950947, CC77DRAFT_1015189, and CC77DRAFT_687848, was upregulated at 6 h and 12 h and downregulated at 24 h and 48 h of treatment (Figure 6), suggesting a potential role in fungal cell wall integrity. These results demonstrated that crude protein extracts from XJ5 significantly impact the transcriptional profile of *A. solani*, especially the genes associated with cell wall synthesis and cell wall integrity. qRT-PCR results of nine randomly selected DEGs correlated well with the transcriptome data (Figure 7). The Pearson correlation coefficient was 0.7465 (Figure 7J), indicating a strong correlation between qRT-PCR results and the transcriptome data.

## 4. Discussion

*B. amyloliquefaciens* is a nonpathogenic bacterium known for its biological control characteristics, including colonization ability, the inhibition of pathogens, and the induction of systemic resistance in plants [33]. While many reports exist on the biocontrol potential of *B. amyloliquefaciens* against diverse phytopathogens [50,51,52,53], there have been limited studies on its effectiveness in reducing the growth of *A. solani*. Herein, we isolated and identified *B. amyloliquefaciens* strain XJ5. In vitro experiments indicated that XJ5 exhibits potent growth inhibition effects on *A. solani*, the causal agent of early blight of tomato, making it a promising biocontrol agent. *B. amyloliquefaciens* strains evidently secrete some antifungal proteins or lipopeptides [15,54]. Similarly, crude protein extracts from XJ5 are known to inhibit mycelial growth and conidia germination and disrupt the hyphal structure of *A. solani* (Figure 1E and Figure 2B–E). These results demonstrated that crude protein extracts of XJ5 have significant antifungal activity; however, the characteristics and mechanisms of crude protein extracts require further exploration.

The complete genome of *B. amyloliquefaciens* offers new insights pertaining to metabolites with potential biocontrol activity [33,55]. Strain FZB42, for example, has an impressive capability to synthesize various secondary metabolites, with approximately 8% of its genome dedicated to antimicrobial metabolite synthesis, while genes related to antimicrobial syntheses in *B. subtilis* account for approximately 4–5% of its genome on average [7,33,56]. Moreover, gene clusters associated with antibacterial substance synthesis have been identified in the genomes of *B. amyloliquefaciens* AS43.3 and DSM7^T^, providing valuable information to unveil the molecular mechanisms of antimicrobial activity in *B. amyloliquefaciens* [28,34]. To elucidate the potential biocontrol mechanism of XJ5, we annotated and analyzed its whole genome sequence. Thirteen BCGs were consequently identified, which included seven gene clusters coding for proteins with amino acid similarities of >90% to seven known classes of antibiotics (surfactin, macrolactin, bacillaene, fengycin, difficidin, bacillibactin, and bacilysin), two synthesis gene clusters with amino acid similarities of <30% to known classes (locillomycin and butirosin A/B), and four functional unknown gene clusters. These well-characterized antibiotics possess broad-spectrum antifungal or antibacterial activity [57,58]. Furthermore, the genome of XJ5 was found to contain genes encoding hydrolases, such as chitinase, xylanase, phosphatase, and protease, which play a vital role in degrading the main components of fungal cell walls (e.g., chitin and chitosan). Our results suggested that the genome of XJ5 encodes several secondary metabolites (lipopeptides and antifungal compounds) and antifungal proteins such as hydrolytic enzymes, which may endow XJ5 with the ability to produce different antifungal substances for biocontrol. Indeed, our biocontrol experiment results indicated that XJ5 can inhibit the growth of *A. solani*, and SEM observations demonstrated that the hyphal cell walls of *A. solani* were deformed and degraded after treatment with XJ5 crude protein extracts (Figure 2). These findings are likely related to the production of different secondary metabolites and cell wall degradation enzymes.

The transcriptome analysis results indicated that many DEGs were enriched in all four stages of treatment; rude protein extracts from XJ5 were observed to significantly impact the transcriptional profile of *A. solani*. KEGG and GO enrichment analyses revealed more upregulated GO terms and KEGG pathways at the early stages (6 and 12 h) of treatment. At this time, the mycelium of *A. solani* was swollen, which coincided with the appearance of spherical vesicles (Figure 2B,C). After 48 h of treatment, the expression of most DEGs was downregulated and hyphal cell walls appeared rough and shriveled, indicating that hyphal cells may be in a stressed physiological metabolic state.

Filamentous fungal cell walls comprise galactomannans, chitin, and β-1,3-glucans, which play an important role in fungal viability and pathogenicity [59]. *Bacillus* spp. can produce various antifungal substances that target fungal cell walls and membranes and then inhibit fungal growth [15,60,61,62]. The expression of genes involved in cell wall synthesis, such as chitin synthase and glycosylation modification-related genes, was inhibited by XJ5 crude protein extracts. The SEM observation indicated that the crude protein extracts from XJ5 can cause hyphal damage and even degradation, these results demonstrated that the hyphal damage may be associated with the downregulation of chitin and mannose synthase-related genes. Furthermore, genes involved in the O-mannosylation (Afpmt1) and N glycan-processing pathways (Afcwh41 and Afams1) can cause deficiencies in cell wall integrity in *Aspergillus fumigatus* [63,64,65,66]. Glutathione S-transferases are apparently involved in protecting cells against damage caused by oxidative stress in *Schizosaccharomyces pombe* and *Saccharomyces cerevisiae* [67,68]. The expression of related genes was upregulated at 6 h and 12 h and downregulated at 24 h and 48 h of treatment. We believe that *A. solani* upregulates the expression of related genes to alleviate stress when exposed to antifungal substances, but the accumulation of these substances ultimately inhibits its growth. XJ5 could disrupt cell wall synthesis and affect the cell wall integrity of *A. solani*. *A. solani* also responds to stress by regulating multiple pathways and gene expression to mitigate the pressure of antifungal substances. To summarize, an antagonistic *B. amyloliquefaciens* strain XJ5 was identified and its biocontrol mechanism against the phytopathogen *A. solani* was investigated by genome and transcriptome sequencing. Our findings should further our understanding of the interactions between *B. amyloliquefaciens* and phytopathogens.

## Figures and Tables

**Table 1:** Secondary metabolite biosynthetic gene clusters.

Cluster ID	Type	Similar Cluster	Similarity %	Number of Genes
Cluster1	NRPS	Locillomycin	28	46
Cluster2	NRPS	Surfactin	91	43
Cluster3	PKS-like	Butirosin A/ButirosinB	7	39
Cluster 4	Terpene	-		23
Cluster5	TransAT-PKS	Macrolactin H	100	43
Cluster6	TransAT-PKS	Bacillaene	100	44
Cluster7	NRPS	Fengycin	100	67
Cluster8	Terpene	-	-	21
Cluster9	T3PKS	-	-	62
Cluster10	TransAT-PKS	Difficidin	100	41
Cluster11	NRPS	Bacillibactin	100	48
Cluster12	NRPS	-	-	38
Cluster13	Other	Bacilysin	100	43

NRPS: Nonribosomal peptide synthetase; PKS-like: other types of polyketide synthases (PKS); transAT-PKS: trans-acyltransferase PKS; T3PKS: type III polyketide synthase; “-” indicates that no synthetic genes with similarity of >1% were found.

## Data Availability

The RNA-Seq data are available at the NCBI Sequence Read Archive (SRA) under BioProject PRJNA989022 (SRA accession number SRR25065848–SRR25065862, sample accession numbers, SAMN36035995–SAMN36036009).

## Supplementary Materials

The following supporting information can be downloaded from: https://www.mdpi.com/article/10.3390/microorganisms11082055/s1; Figure S1: KEGG annotation classification (A) and enzyme annotation in the CAZy database (B) of *B. amyloliquefaciens* XJ5; Table S1: Genomic features of XJ5; Table S2: Primers used for qRT-PCR; Table S3: Statistics of transcriptional group sequencing data; and Table S4: The information of genes in the thirteen clusters.
