# Supplementary material for: Genome and Transcriptome Analysis to Elucidate the Biocontrol Mechanism of Bacillus amyloliquefaciens XJ5 against Alternaria solani
Source: Microorganisms. 2023 Aug 10;11(8):2055. doi: 10.3390/microorganisms11082055 (PMC10459136; doi:10.3390/microorganisms11082055)
Supplement: Supplementary file 1 [file microorganisms-11-02055-s001.zip › microorganisms-2529359-supplementary.pdf]

Journal of *Fungi*

Supplementary Information: Genome and Transcriptome Analysis to Elucidate the Biocontrol Mechanism of *Bacillus amyloliquefaciens* XJ5 against *Alternaria solani*

Fan Mu, Xu Chen, Zhenxin Fu, Xue Wang, Jiexin Guo, Xiaojun Zhao and Baojun Zhang *

Shanxi Key Laboratory of Integrated Pest Management in Agriculture, College of Plant Protection, Shanxi Agricultural University, Jinzhong 030801, China; fanmu1993@126.com (F.M.); cx15294780353@163.com (X.C.); s20212327@stu.sxau.edu.cn (Z.F.); 17866709612@163.com (X.W.); 15234483340@163.com (J.G.); zhaoxiaojun0218@163.com (X.Z.)

* Correspondence: sxauzbj@sxau.edu.cn; Tel: +86-0354-628-5015

Figure S1. KEGG annotation classification (A) and enzymes annotation in the CAZy database (B) of *B. amyloliquefaciens* XJ5.

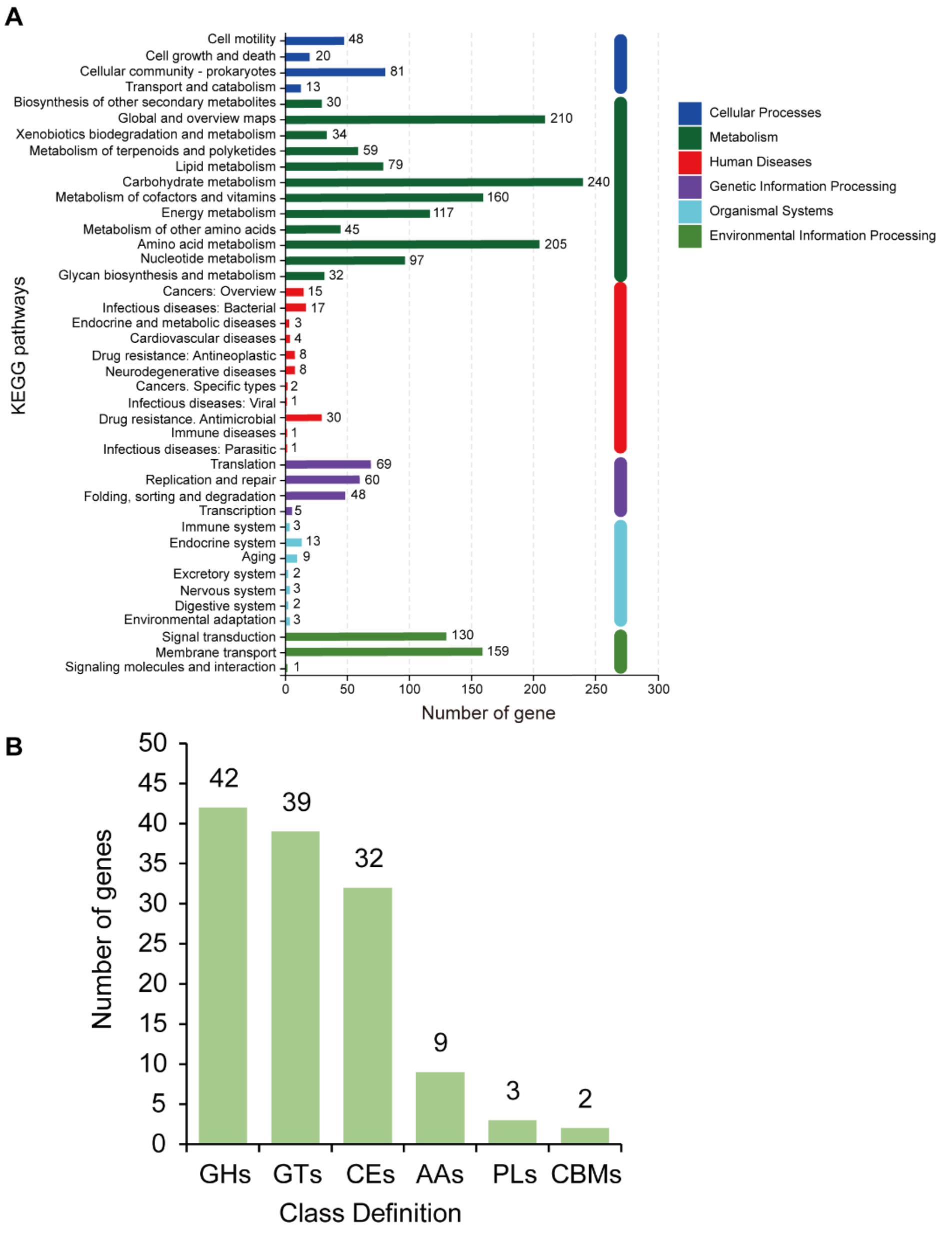

Table S1. Primers used for qRT-PCR.

Gene ID	Primer sequence (5' to 3')
Actin (CC77DRAFT_1021406)	F：GACATGGCTGGTCGTGAT
	R：AGTGGTGGAGAAGGTGTAAC
CC77DRAFT_1056386	F：AAGCCGATGTCGTTAGCCT
	R：TCGTCCAAATCCAGACTCCT
CC77DRAFT_1015889	F：GTCAACTGCGGGTGGTCA
	R：GTATGCGCTGCACGTATCG
CC77DRAFT_752966	F：AACCGCGTCGTAAGCATCCT
	R：TCGCACAGTATCGCCAGTCT
CC77DRAFT_1061421	F：GACAACACCACCGCCTTACC
	R：CCACTGGCGTTGATGAGCT
CC77DRAFT_1032488	F：CCATAACTGGCGTCTCATCTAC
	R：TCAATCTGACTCCTATTCTGC
CC77DRAFT_1032980	F：CACGATGGGAATACCGCAAG
	R：GGATCATGGAGGTGACGAACG
CC77DRAFT_973065	F：TCTCGGGATACCGTGCCATT
	R：CGCTTGTTTCGCCTTCTTCGT
CC77DRAFT_1067874	F：CATTATCGGCTGCAGTTGCG
	R：GATTCCGGCGTCATTCCTCT
CC77DRAFT_687848	F：TCCTGCGCTCACAGATACCT
	R：TAGCTTGACCTTGCATGGGC

Table S2. Genomic features of the *B. amyloliquefaciens* XJ5

Features	Genome
Genome size (bp)	4,160,003
G+C content (%)	46.09
Protein-coding genes	4199
Gene total length (bp)	3,939,753
Gene/genome (%)	88.74
Intergenetic region length (bp)	499,752
Intergenetic length/genome (%)	11.26
5S rRNA	9
16S rRNA	9
23S rRNA	9
tRNA	88

Table S3. Statistics of transcriptional group sequencing data

Sample	Raw reads	Clean reads	Q20(%)	Q30(%)	Mapped Reads
CK-1	47923348	47437924	98.96	96.57	39,980,345(84.28%)
CK-2	44675094	44252032	98.93	96.50	37,381,401(84.47%)
CK-3	49962588	49456594	98.92	96.46	41,421,266(83.75%)
EP6h-1	52190462	51665380	98.96	96.56	42,504,386(82.27%)
EP6h-2	49152032	48617778	98.96	96.59	41,470,091(85.3%)
EP6h-3	49277010	48777482	98.89	96.39	42,420,783(86.97%)
EP12h-1	53502866	52970852	98.91	96.42	45,575,068(86.04%)
EP12h-2	54218470	53717634	98.92	96.44	46,151,893(85.92%)
EP12h-3	49520036	49092778	98.87	96.30	41,867,157(85.28%)
EP24h-1	50111002	49605948	98.88	96.37	42,882,097(86.45%)
EP24h-2	50011662	49564260	98.97	96.60	42,245,634(85.23%)
EP24h-3	49505922	49061604	98.91	96.42	42,450,098(86.52%)
EP48h-1	49886078	49364242	98.98	96.63	42,862,509(86.83%)

Table S4. The information of genes in the thirteen clusters

Cluster 1				
**Table of genes**	**loca-tions**	**loca-tions**	**strands**	**annotations of query cluster**
J4048_01135	197903	199250	+	phosphoglucosamine mutase
J4048_01140	199681	201484	+	glutamine--fructose-6-phosphate transaminase (isomerizing)
J4048_01145	201902	202775	+	ABC transporter ATP-binding protein
J4048_01150	202752	203406	+	ABC-2 transporter permease
J4048_01155	203508	204390	-	hypothetical protein
J4048_01160	204524	205691	+	DUF4885 family protein
J4048_01165	205743	206589	-	aldo/keto reductase
J4048_01170	206699	207392	-	LrgB family protein
J4048_01175	207361	207766	-	CidA/LrgA family holin-like protein
J4048_01180	207961	208393	+	MarR family transcriptional regulator
J4048_01185	208819	210205	+	amino acid permease
J4048_01190	210297	211002	+	YjjG family noncanonical pyrimidine nucleotidase
J4048_01195	211057	212215	+	sensor histidine kinase
J4048_01200	212238	212892	+	response regulator transcription factor
J4048_01205	213109	214042	+	ABC transporter ATP-binding protein
J4048_01210	214057	215266	+	ABC transporter permease
J4048_01215	215268	216435	+	ABC transporter permease
J4048_01220	216484	216664	+	hypothetical protein
J4048_01225	216736	217432	+	thioesterase
J4048_01230	217804	225427	+	non-ribosomal peptide synthetase
J4048_01235	225386	233057	+	amino acid adenylation domain-containing protein
J4048_01240	233016	243774	+	amino acid adenylation domain-containing protein
J4048_01245	243856	245110	+	ACP S-malonyltransferase
J4048_01250	245153	249926	+	SDR family NAD(P)-dependent oxidoreductase
J4048_01255	250111	254902	+	SDR family NAD(P)-dependent oxidoreductase
J4048_01260	254923	256447	+	NAD(P)/FAD-dependent oxidoreductase
J4048_01265	256544	263165	+	non-ribosomal peptide synthetase
J4048_01270	263185	264229	+	helix-turn-helix domain-containing protein
J4048_01275	264385	264862	+	DUF4879 domain-containing protein
J4048_01280	264942	265191	+	sigma-G-dependent sporulation-specific acid-soluble spore protein CsgA
J4048_01285	265300	265999	+	DUF308 domain-containing protein
J4048_01290	266043	266304	-	hypothetical protein
J4048_01295	266489	268109	+	APC family permease
J4048_01300	268512	269220	+	hypothetical protein
J4048_01305	269281	270484	+	hypothetical protein
J4048_01310	270558	270807	+	DUF2651 family protein
J4048_01315	270984	271872	-	glycerophosphodiester phosphodiesterase
J4048_01320	271967	273302	-	glycerol-3-phosphate transporter
J4048_01325	273636	273879	+	DUF2651 family protein
J4048_01330	273934	275380	-	4-hydroxyphenylacetate 3-monooxygenase, oxygenase component
J4048_01335	275530	275956	-	hypothetical protein
J4048_01340	276028	278203	-	DUF1906 domain-containing protein
J4048_01345	278578	279022	+	hypothetical protein
J4048_01350	279284	279755	+	helix-turn-helix domain-containing protein
J4048_01355	279826	280897	+	iron-containing alcohol dehydrogenase family protein
J4048_01360	280978	282133	+	phosphoribosylglycinamide formyltransferase 2

J4048_01365	282609	283143	+	CDP-diacylglycerol--serine O-phosphatidyltransferase
**Cluster 2**				
**Table of genes**	**loca-tions**	**loca-tions**	**strands**	**annotations of query cluster**
J4048_01750	361736	362747	+	NAD(P)/FAD-dependent oxidoreductase
J4048_01755	362802	364242	-	uroporphyrinogen-III C-methyltransferase
J4048_01760	364308	364629	-	nitrite reductase small subunit NirD
J4048_01765	364649	367067	-	NADPH-nitrite reductase
J4048_01770	367186	369319	-	nitrate reductase
J4048_01775	369311	371639	-	FAD-dependent oxidoreductase
J4048_01780	371819	373025	+	NarK/NasA family nitrate transporter
J4048_01785	373212	374415	+	GTP-binding protein
J4048_01790	374479	375616	+	glutathione-dependent formaldehyde dehydrogenase
J4048_01795	375630	376065	+	RDD family protein
J4048_01800	376136	376460	+	YckD family protein
J4048_01805	376563	378000	+	family 1 glycosylhydrolase
J4048_01810	378040	378439	-	competence protein ComJ
J4048_01815	378459	378897	-	sporulation protein
J4048_01820	379254	379812	-	6-phospho-3-hexuloisomerase
J4048_01825	379808	380444	-	3-hexulose-6-phosphate synthase
J4048_01830	380675	381038	+	helix-turn-helix transcriptional regulator
J4048_01835	381626	392381	+	surfactin non-ribosomal peptide synthetase SrfAA
J4048_01840	392402	403163	+	surfactin non-ribosomal peptide synthetase SrfAB
J4048_01845	403197	407034	+	surfactin non-ribosomal peptide synthetase SrfAC
J4048_01850	407053	407785	+	surfactin biosynthesis thioesterase SrfAD
J4048_01855	407906	409217	+	aminotransferase class I/II-fold pyridoxal phosphate-dependent enzyme
J4048_01860	409251	409791	-	YcxB family protein
J4048_01865	409787	410750	-	DMT family transporter
J4048_01870	410868	412179	+	PLP-dependent aminotransferase family protein
J4048_01875	412173	412848	-	4'-phosphopantetheinyl transferase superfamily protein
J4048_01880	412945	413575	-	YitT family protein
J4048_01885	413674	414418	-	cystine ABC transporter ATP-binding protein TcyC
J4048_01890	414430	415135	-	amino acid ABC transporter permease
J4048_01895	415121	415919	-	cystine ABC transporter substrate-binding lipoprotein TcyA
J4048_01900	416047	416920	-	LysR family transcriptional regulator
J4048_01905	417013	417628	+	UbiX family flavin prenyltransferase
J4048_01910	417630	419052	+	phenolic acid decarboxylase BsdC
J4048_01915	419069	419297	+	hypothetical protein
J4048_01920	419296	419758	+	pyridoxamine 5'-phosphate oxidase family protein
J4048_01925	419806	421285	-	peptide MFS transporter
J4048_01930	421563	423309	+	right-handed parallel beta-helix repeat-containing protein
J4048_01935	423400	423622	-	hypothetical protein
J4048_01940	423743	425384	+	spore germination protein
J4048_01945	425370	426597	+	Ger(x)C family spore germination protein
**Cluster 3**				
**Table of genes**	**loca-tions**	**loca-tions**	**strands**	**annotations of query cluster**
J4048_04825	963295	963730	-	nitric oxide-sensing transcriptional repressor NsrR
J4048_04830	963864	965379	-	mechanosensitive ion channel
J4048_04835	965548	966955	+	SpoVR family protein

J4048_04840	966993	968373	-	alkaline phosphatase
J4048_04845	968831	969656	+	LysM peptidoglycan-binding domain-containing protein
J4048_04850	969724	970600	-	LysR family transcriptional regulator
J4048_04855	970709	971813	+	citrate synthase/methylcitrate synthase
J4048_04860	971892	972762	+	SDR family oxidoreductase
J4048_04865	973092	974496	+	amino acid permease
J4048_04870	974610	975966	+	sodium-dependent transporter
J4048_04875	976002	976293	-	anti-sigma factor
J4048_04880	976280	977354	-	anti-sigma factor
J4048_04885	977346	977838	-	RNA polymerase sigma factor SigM
J4048_04890	978078	978678	+	1-acylglycerol-3-phosphate O-acyltransferase
J4048_04895	978723	980058	-	HlyC/CorC family transporter
J4048_04900	980119	980545	-	MerR family transcriptional regulator
J4048_04905	980724	981909	+	pyridoxal phosphate-dependent aminotransferase
J4048_04910	981941	983186	+	polyketide beta-ketoacyl:ACP synthase
J4048_04915	983415	984804	+	HlyC/CorC family transporter
J4048_04920	984817	985177	-	fluoride efflux transporter CrcB
J4048_04925	985173	985569	-	CrcB family protein
J4048_04930	985570	986287	-	glycerophosphodiester phosphodiesterase
J4048_04935	986456	986561	+	YhdX family protein
J4048_04940	986723	987839	+	mechanosensitive ion channel family protein
J4048_04945	987890	988634	+	NAD-dependent protein deacylase
J4048_04950	988667	989516	-	polysaccharide deacetylase
J4048_04955	989772	990624	+	D-amino-acid transaminase
J4048_04960	990665	991250	-	TetR/AcrR family transcriptional regulator
J4048_04965	991358	991910	+	hypothetical protein
J4048_04970	991948	992449	-	universal stress protein
J4048_04975	992870	994628	+	ATP-binding cassette domain-containing protein
J4048_04980	994624	996646	+	ABC transporter ATP-binding protein
J4048_04985	996685	997306	-	NAD(P)H-binding protein
J4048_04990	997561	997768	-	alpha/beta-type small acid-soluble spore protein
J4048_04995	997992	998208	-	YheE family protein
J4048_05000	998290	999649	-	YheC/YheD family protein
J4048_05005	999638	1000730	-	YheC/YheD family protein
J4048_05010	1000967	1002101	+	DUF445 domain-containing protein
J4048_05015	1002186	1002537	+	YlbF family regulator
**Cluster 4**				
**Table of genes**	**loca-tions**	**loca-tions**	**strands**	**annotations of query cluster**
J4048_05435	1088790	1089084	+	HNH endonuclease
J4048_05440	1089127	1089346	-	spore germination protein
J4048_05445	1089385	1089781	-	spore germination protein GerPE
J4048_05450	1089780	1089957	-	spore gernimation protein GerPD
J4048_05455	1089953	1090571	-	spore germination protein GerPC
J4048_05460	1090606	1090843	-	spore gernimation protein GerPB
J4048_05465	1090855	1091077	-	spore germination protein
J4048_05470	1091266	1091482	-	aspartyl-phosphate phosphatase Spo0E family protein
J4048_05475	1091699	1092605	+	fumarylacetoacetate hydrolase family protein
J4048_05480	1092698	1093055	+	YisL family protein
J4048_05485	1093055	1093273	-	hypothetical protein

J4048_05490	1093308	1093851	-	DUF2777 family protein
J4048_05495	1094026	1095868	+	asparagine synthase (glutamine-hydrolyzing)
J4048_05500	1095953	1096694	+	squalene/phytoene synthase family protein
J4048_05505	1096728	1098096	-	MATE family efflux transporter
J4048_05510	1098218	1099082	+	helix-turn-helix transcriptional regulator
J4048_05515	1099225	1099720	-	damage-inducible protein DinB
J4048_05520	1099773	1100382	-	amino acid transporter
J4048_05525	1100507	1101950	+	PLP-dependent aminotransferase family protein
J4048_05530	1101957	1102584	-	pentapeptide repeat-containing protein
J4048_05535	1102779	1103220	-	GNAT family N-acetyltransferase
J4048_05540	1103302	1105144	-	bifunctional homocysteine S-methyltransferase/methylenetetrahydrofolate reductase
J4048_05545	1105455	1105947	-	YajQ family cyclic di-GMP-binding protein
**Cluster 5**				
**Table of genes**	**locations**	**locations**	**strands**	**annotations of query cluster**
J4048_07285	1421706	1422618	+	1-phosphofructokinase
J4048_07290	1422632	1424537	+	PTS sugar transporter subunit IIA
J4048_07295	1424667	1425249	+	signal peptidase I
J4048_07300	1425285	1425555	-	hypothetical protein
J4048_07305	1425717	1427337	+	ATP-binding cassette domain-containing protein
J4048_07310	1427449	1428358	+	ketopantoate reductase family protein
J4048_07315	1428389	1429622	-	aminopeptidase
J4048_07320	1429722	1429857	-	protein YkpC
J4048_07325	1429928	1430936	-	cell shape-determining protein MreBH
J4048_07330	1431206	1431491	+	AbrB/MazE/SpoVT family DNA-binding domain-containing protein
J4048_07335	1431663	1432968	+	PAS domain-containing protein
J4048_07340	1432969	1433797	+	gamma-glutamylcyclotransferase
J4048_07345	1433837	1434503	+	Ktr system potassium transporter KtrC
J4048_07350	1434651	1436385	+	adenine deaminase
J4048_07355	1436417	1438085	-	ribonuclease J1
J4048_07360	1438090	1438300	-	DNA-dependent RNA polymerase auxiliary subunit epsilon family protein
J4048_07365	1438654	1439428	+	Cof-type HAD-IIB family hydrolase
J4048_07370	1439462	1440017	-	peptide deformylase
J4048_07375	1440129	1440309	-	YjcZ family sporulation protein
J4048_07380	1440553	1441192	+	YkyA family protein
J4048_07385	1441647	1443954	+	ACP S-malonyltransferase
J4048_07390	1443975	1456230	+	SDR family NAD(P)-dependent oxidoreductase
J4048_07395	1456229	1461002	+	SDR family NAD(P)-dependent oxidoreductase
J4048_07400	1461049	1469758	+	SDR family NAD(P)-dependent oxidoreductase
J4048_07405	1469750	1476755	+	SDR family NAD(P)-dependent oxidoreductase
J4048_07410	1476778	1482490	+	SDR family NAD(P)-dependent oxidoreductase
J4048_07415	1482489	1489869	+	SDR family NAD(P)-dependent oxidoreductase
J4048_07420	1489919	1493771	+	alpha/beta fold hydrolase
J4048_07425	1493803	1494895	+	beta-lactamase family protein
J4048_07430	1495365	1496481	+	pyruvate dehydrogenase (acetyl-transferring) E1 component subunit alpha
J4048_07435	1496484	1497462	+	pyruvate dehydrogenase complex E1 component subunit beta
J4048_07440	1497585	1498914	+	pyruvate dehydrogenase complex dihydrolipoyllysine-residue acetyltransferase
J4048_07445	1498918	1500331	+	dihydrolipoyl dehydrogenase
J4048_07450	1500378	1500753	-	hypothetical protein

J4048_07455	1501001	1501802	+	polysaccharide deacetylase family protein
J4048_07460	1501915	1502035	+	GapA-binding peptide SR1P
J4048_07465	1502082	1503555	-	aminotransferase class I/II-fold pyridoxal phosphate-dependent enzyme
J4048_07470	1503734	1504001	+	UPF0223 family protein
J4048_07475	1504035	1504668	-	DUF1054 domain-containing protein
J4048_07480	1504904	1505093	+	hypothetical protein
J4048_07485	1505226	1506024	+	inositol-1-monophosphatase
J4048_07490	1506010	1506463	+	GNAT family N-acetyltransferase
J4048_07495	1506514	1508080	-	peptidase M4 family protein
**Cluster 6**				
**Table of genes**	**loca-tions**	**loca-tions**	**strands**	**annotations of query cluster**
J4048_08615	1735555	1737115	+	ribonuclease Y
J4048_08620	1737175	1737970	+	TIGR00282 family metallophosphoesterase
J4048_08625	1738169	1738430	+	stage V sporulation protein SpoVS
J4048_08630	1738687	1739734	+	L-threonine 3-dehydrogenase
J4048_08635	1739746	1740925	+	glycine C-acetyltransferase
J4048_08640	1741067	1742597	+	tRNA (N6-isopentenyl adenosine(37)-C2)-methylthiotransferase MiaB
J4048_08645	1742599	1743031	+	RicAFT regulatory complex protein RicA family protein
J4048_08650	1743285	1743831	+	outer spore coat protein CotE
J4048_08655	1743949	1746535	+	DNA mismatch repair protein MutS
J4048_08660	1746550	1748425	+	DNA mismatch repair endonuclease MutL
J4048_08665	1748476	1749094	-	site-specific integrase
J4048_08670	1749532	1749835	+	hypothetical protein
J4048_08675	1750870	1751080	-	hypothetical protein
J4048_08680	1751166	1751688	-	hypothetical protein
J4048_08685	1753220	1753898	+	MBL fold metallo-hydrolase
J4048_08690	1754212	1755082	+	ACP S-malonyltransferase
J4048_08695	1755218	1756193	+	acyltransferase domain-containing protein
J4048_08700	1756194	1758435	+	ACP S-malonyltransferase
J4048_08705	1758500	1758749	+	acyl carrier protein
J4048_08710	1758800	1760063	+	hydroxymethylglutaryl-CoA synthase family protein
J4048_08715	1760059	1760833	+	enoyl-CoA hydratase/isomerase
J4048_08720	1760842	1761592	+	enoyl-CoA hydratase/isomerase family protein
J4048_08725	1761631	1776583	+	non-ribosomal peptide synthetase
J4048_08730	1776584	1789991	+	SDR family NAD(P)-dependent oxidoreductase
J4048_08735	1790008	1800556	+	SDR family NAD(P)-dependent oxidoreductase
J4048_08740	1800545	1816844	+	non-ribosomal peptide synthetase
J4048_08745	1816857	1824315	+	polyketide synthase dehydratase domain-containing protein
J4048_08750	1824451	1825663	-	cytochrome P450
J4048_08755	1825951	1826386	+	sporulation protein
J4048_08760	1826445	1827201	+	DUF1275 domain-containing protein
J4048_08765	1827234	1827597	-	hypothetical protein
J4048_08770	1827789	1829118	-	S8 family peptidase
J4048_08775	1829296	1829530	+	hypothetical protein
J4048_08780	1829785	1830493	+	poly-gamma-glutamate hydrolase family protein
J4048_08785	1830552	1831005	+	OsmC family protein
J4048_08790	1831018	1831375	-	multidrug efflux SMR transporter
J4048_08795	1831389	1831704	-	multidrug efflux SMR transporter
J4048_08800	1831841	1832147	-	hypothetical protein

J4048_08805	1832248	1832653	+	hypothetical protein
J4048_08810	1832751	1833696	+	tRNA (adenosine(37)-N6)-dimethylallyltransferase MiaA
J4048_08815	1833735	1833957	+	RNA chaperone Hfq
J4048_08820	1834053	1834329	+	hypothetical protein
J4048_08825	1834411	1834627	+	hypothetical protein
J4048_08830	1834886	1835279	+	class Ib ribonucleoside-diphosphate reductase assembly flavoprotein NrdI
**Cluster 7**				
**Table of genes**	**loca-tions**	**loca-tions**	**strands**	**annotations of query cluster**
J4048_09255	1915060	1915279	+	hypothetical protein
J4048_09260	1915586	1915727	+	hypothetical protein
J4048_09265	1916228	1916831	+	hypothetical protein
J4048_09270	1916952	1917288	-	hypothetical protein
J4048_09275	1918051	1919158	+	tetratricopeptide repeat protein
J4048_09280	1919154	1919295	+	hypothetical protein
J4048_09285	1919467	1919746	+	hypothetical protein
J4048_09290	1919909	1920575	-	DUF2325 domain-containing protein
J4048_09295	1920887	1921247	+	lactococcin 972 family bacteriocin
J4048_09300	1921332	1923498	+	DUF1430 domain-containing protein
J4048_09305	1923494	1924115	+	ABC transporter ATP-binding protein
J4048_09310	1924128	1924338	+	bacteriocin-like WGxF protein
J4048_09315	1924596	1924908	+	hypothetical protein
J4048_09320	1925199	1926061	-	choline esterase
J4048_09325	1926318	1927818	-	cellulase family glycosylhydrolase
J4048_09330	1928267	1928579	+	DUF4870 domain-containing protein
J4048_09335	1928630	1930031	-	HAMP domain-containing histidine kinase
J4048_09340	1930033	1930741	-	response regulator transcription factor
J4048_09345	1930882	1932154	-	glucuronoxylanase
J4048_09350	1932231	1933770	-	carbohydrate-binding protein
J4048_09355	1934094	1941951	-	non-ribosomal peptide synthetase
J4048_09360	1942039	1958128	-	non-ribosomal peptide synthetase
J4048_09365	1958172	1970121	-	bacillomycin D hybrid PKS/NRPS BamA
J4048_09370	1970140	1971343	-	bacillomycin D biosynthesis malonyl-CoA transacylase BamD
J4048_09375	1971897	1972683	-	3-hydroxybutyrate dehydrogenase
J4048_09380	1972695	1973358	-	CoA transferase subunit B
J4048_09385	1973375	1974077	-	CoA transferase subunit A
J4048_09390	1974101	1975538	-	GntP family permease
J4048_09395	1975840	1977037	-	cytochrome P450
J4048_09400	1977038	1978058	-	biotin synthase BioB
J4048_09405	1978060	1978762	-	ATP-dependent dethiobiotin synthetase BioD
J4048_09410	1978758	1979919	-	8-amino-7-oxononanoate synthase
J4048_09415	1979908	1981255	-	adenosylmethionine--8-amino-7-oxononanoate transaminase
J4048_09420	1981251	1982022	-	6-carboxyhexanoate--CoA ligase
J4048_09425	1982298	1982706	+	GtrA family protein
J4048_09430	1982712	1983606	+	UTP--glucose-1-phosphate uridylyltransferase GalU
J4048_09435	1983680	1984271	+	DedA family protein
J4048_09440	1984324	1985854	-	acyl-CoA carboxylase subunit beta
J4048_09445	1985871	1986651	-	enoyl-CoA hydratase
J4048_09450	1986664	1987564	-	hydroxymethylglutaryl-CoA lyase
J4048_09455	1987579	1987792	-	acetyl-CoA carboxylase biotin carboxyl carrier protein subunit

J4048_09460	1987788	1989138	-	acetyl-CoA carboxylase biotin carboxylase subunit
J4048_09465	1989159	1990800	-	AMP-binding protein
J4048_09470	1990845	1991988	-	acyl-CoA dehydrogenase family protein
J4048_09475	1992128	1993667	-	family 10 glycosylhydrolase
J4048_09480	1993789	1994170	-	DUF1360 domain-containing protein
J4048_09485	1994242	1998046	-	non-ribosomal peptide synthase
J4048_09490	1998064	2008840	-	amino acid adenylation domain-containing protein
J4048_09495	2008865	2016515	-	non-ribosomal peptide synthetase
J4048_09500	2016530	2024228	-	non-ribosomal peptide synthetase
J4048_09505	2024253	2031933	-	amino acid adenylation domain-containing protein
J4048_09510	2031948	2039647	-	non-ribosomal peptide synthetase
J4048_09515	2039672	2047331	-	non-ribosomal peptide synthetase
J4048_09520	2047810	2049286	-	D-alanyl-D-alanine carboxypeptidase/D-alanyl-D-alanine-endopeptidase
J4048_09525	2049304	2050273	-	aldose 1-epimerase
J4048_09530	2050388	2051777	-	MATE family efflux transporter
J4048_09535	2052003	2052546	+	IseA DL-endopeptidase inhibitor family protein
J4048_09545	2052979	2053525	-	site-specific integrase
J4048_09550	2053839	2054070	-	excisionase family DNA-binding protein
J4048_09555	2054313	2056089	+	gamma-glutamyltransferase
J4048_09560	2056133	2057309	-	MFS transporter
J4048_09565	2057452	2057755	-	winged helix-turn-helix transcriptional regulator
J4048_09570	2057801	2058086	-	threonyl-tRNA synthetase
J4048_09575	2058085	2058949	-	LysR family transcriptional regulator
J4048_09580	2059067	2060048	+	zinc-binding dehydrogenase
J4048_09585	2060371	2061853	-	glutamate synthase small subunit
J4048_09590	2061869	2066429	-	glutamate synthase large subunit
**Cluster 8**				
**Table of genes**	**loca-tions**	**loca-tions**	**strands**	**annotations of query cluster**
J4048_09730	2090607	2091000	-	hypothetical protein
J4048_09735	2091162	2092032	-	LysM peptidoglycan-binding domain-containing protein
J4048_09740	2092267	2094046	-	DNA helicase RecQ
J4048_09750	2094554	2095181	-	FMN-dependent NADH-azoreductase
J4048_09755	2095325	2095817	-	TraR/DksA C4-type zinc finger protein
J4048_09760	2095899	2096280	-	hypothetical protein
J4048_09765	2096372	2096606	+	hypothetical protein
J4048_09770	2096592	2097075	-	Hsp20/alpha crystallin family protein
J4048_09775	2097145	2097418	+	hypothetical protein
J4048_09780	2097414	2097648	+	hypothetical protein
J4048_09785	2097692	2098037	-	PH domain-containing protein
J4048_09790	2098330	2098471	-	DUF4025 domain-containing protein
J4048_09795	2098536	2098740	-	hypothetical protein
J4048_09800	2098923	2100411	+	aldehyde dehydrogenase family protein
J4048_09805	2100506	2102391	+	squalene--hopene cyclase
J4048_09810	2102387	2103233	+	superoxide dismutase
J4048_09815	2103272	2104610	-	sodium-dependent transporter
J4048_09820	2104809	2105781	+	bile acid:sodium symporter family protein
J4048_09825	2105813	2106560	-	MBL fold metallo-hydrolase
J4048_09830	2106632	2107880	-	2-oxoglutarate dehydrogenase complex dihydrolipoyllysine-residue succinyl-transferase

J4048_09835	2107895	2110730	-	2-oxoglutarate dehydrogenase E1 component
**Cluster 9**				
**Table of genes**	**loca-tions**	**loca-tions**	**strands**	**annotations of query cluster**
J4048_10260	2179462	2179948	-	dihydrofolate reductase
J4048_10265	2179944	2180739	-	thymidylate synthase
J4048_10270	2180838	2181447	-	YpjP family protein
J4048_10275	2181709	2182480	-	class I SAM-dependent methyltransferase
J4048_10280	2182519	2182954	-	BrxA/BrxB family bacilliredoxin
J4048_10285	2183035	2184712	-	dihydroxy-acid dehydratase
J4048_10290	2184964	2186098	-	conserved virulence factor C family protein
J4048_10295	2186166	2186774	-	HD domain-containing protein
J4048_10300	2186785	2187268	-	glutathione peroxidase
J4048_10305	2187551	2188457	+	homoserine O-succinyltransferase
J4048_10310	2188681	2189824	+	diglucosyl diacylglycerol synthase
J4048_10315	2189902	2190029	+	hypothetical protein
J4048_10320	2190044	2190245	+	cold-shock protein CspD
J4048_10325	2190296	2190479	-	hypothetical protein
J4048_10330	2190640	2190898	+	DUF2564 family protein
J4048_10335	2190924	2191107	-	zinc-finger domain-containing protein
J4048_10340	2191109	2191781	-	reverse transcriptase-like protein
J4048_10345	2191862	2192543	+	queuosine precursor transporter
J4048_10350	2192549	2192942	+	reverse transcriptase-like protein
J4048_10355	2192993	2193122	+	small, acid-soluble spore protein L
J4048_10360	2193127	2194015	-	5'-3' exonuclease
J4048_10365	2194110	2194254	-	hypothetical protein
J4048_10370	2194330	2194588	-	YpbS family protein
J4048_10375	2194656	2198238	-	dynamin family protein
J4048_10380	2198421	2198517	-	Fur-regulated basic protein FbpC
J4048_10385	2198589	2199096	-	isoprenylcysteine carboxyl methyltransferase family protein
J4048_10390	2199095	2200196	-	type III polyketide synthase
J4048_10395	2200361	2200970	+	GNAT family N-acetyltransferase
J4048_10400	2200998	2201307	-	MGMT family protein
J4048_10405	2201528	2201792	+	transcriptional regulator
J4048_10410	2202528	2203106	+	DUF421 domain-containing protein
J4048_10415	2203346	2203913	-	cysteine hydrolase
J4048_10420	2204262	2204538	+	hypothetical protein
J4048_10425	2204785	2205706	-	purine permease
J4048_10430	2205741	2205894	-	hypothetical protein
J4048_10435	2205902	2206457	-	Holliday junction resolvase RecU
J4048_10440	2206554	2206794	+	helix-turn-helix transcriptional regulator
J4048_10445	2207024	2207189	-	hypothetical protein
J4048_10450	2207185	2207359	-	hypothetical protein
J4048_10455	2207373	2207691	-	hypothetical protein
J4048_10460	2207787	2208108	-	hypothetical protein
J4048_10465	2208214	2208367	-	hypothetical protein
J4048_10470	2208411	2208651	-	hypothetical protein
J4048_10475	2208689	2208869	-	hypothetical protein
J4048_10480	2208914	2209136	-	hypothetical protein
J4048_10485	2209145	2209973	-	metallophosphoesterase

J4048_10490	2209974	2210919	-	HNH endonuclease
J4048_10495	2211186	2211528	-	hypothetical protein
J4048_10500	2211655	2212018	-	hypothetical protein
J4048_10505	2212014	2212365	-	hypothetical protein
J4048_10510	2212502	2212829	-	hypothetical protein
J4048_10515	2212888	2213254	-	hypothetical protein
J4048_10520	2213382	2213889	-	dihydrofolate reductase
J4048_10525	2213888	2214728	-	thymidylate synthase
J4048_10530	2215503	2215803	-	hypothetical protein
J4048_10535	2215920	2216085	-	hypothetical protein
J4048_10540	2216195	2216810	-	hypothetical protein
J4048_10545	2216868	2217231	-	hypothetical protein
J4048_10550	2217279	2217786	-	hypothetical protein
J4048_10555	2217785	2218034	-	thioredoxin
J4048_10560	2218030	2219026	-	class 1b ribonucleoside-diphosphate reductase subunit beta
J4048_10565	2219089	2219428	+	hypothetical protein
**Cluster 10**				
**Table of genes**	**loca-tions**	**loca-tions**	**strands**	**annotations of query cluster**
J4048_12175	2484067	2484844	-	SDR family oxidoreductase
J4048_12180	2484849	2485809	-	MBL fold metallo-hydrolase
J4048_12185	2486204	2487044	+	pyrroline-5-carboxylate reductase
J4048_12190	2487080	2487827	-	enoyl-CoA hydratase/isomerase family protein
J4048_12195	2487886	2489134	-	hydroxymethylglutaryl-CoA synthase family protein
J4048_12200	2489191	2490346	-	cytochrome P450
J4048_12205	2490427	2496643	-	zinc-binding dehydrogenase
J4048_12210	2496639	2502792	-	SDR family NAD(P)-dependent oxidoreductase
J4048_12215	2502814	2510533	-	SDR family NAD(P)-dependent oxidoreductase
J4048_12220	2510537	2526152	-	SDR family NAD(P)-dependent oxidoreductase
J4048_12225	2526203	2531930	-	SDR family NAD(P)-dependent oxidoreductase
J4048_12230	2531969	2538266	-	KR domain-containing protein
J4048_12235	2538284	2550878	-	SDR family NAD(P)-dependent oxidoreductase
J4048_12240	2550917	2551655	-	SDR family oxidoreductase
J4048_12245	2551669	2553034	-	AMP-binding protein
J4048_12250	2553030	2553303	-	acyl carrier protein
J4048_12255	2553327	2554308	-	D-fructose-6-phosphate amidotransferase
J4048_12260	2554348	2556607	-	ACP S-malonyltransferase
J4048_12265	2557341	2557872	+	antiterminator LoaP
J4048_12270	2557888	2558071	-	hypothetical protein
J4048_12275	2558103	2558316	+	hypothetical protein
J4048_12280	2558455	2559472	+	NADPH dehydrogenase NamA
J4048_12285	2559585	2560359	+	alpha/beta fold hydrolase
J4048_12290	2560408	2560558	-	50S ribosomal protein L33
J4048_12295	2560636	2561563	-	ribonuclease Z
J4048_12300	2561771	2563241	+	glucose-6-phosphate dehydrogenase
J4048_12305	2563330	2564740	-	NADP-dependent phosphogluconate dehydrogenase
J4048_12310	2564848	2566093	-	DNA polymerase IV
J4048_12315	2566168	2566450	+	hypothetical protein
J4048_12320	2566482	2567319	+	membrane protein insertase YidC
J4048_12325	2567432	2568548	-	M20/M25/M40 family metallo-hydrolase

J4048_12330	2568564	2570082	-	acyl-CoA carboxylase subunit beta
J4048_12335	2570074	2570503	-	methylmalonyl-CoA epimerase
J4048_12340	2570596	2570692	+	stressosome-associated protein Prli42
J4048_12345	2570705	2571236	-	L,D-transpeptidase
J4048_12350	2571306	2572260	-	aromatic acid exporter family protein
J4048_12355	2572336	2573059	-	amino acid ABC transporter ATP-binding protein
J4048_12360	2573051	2573711	-	amino acid ABC transporter permease
J4048_12365	2573772	2574540	-	transporter substrate-binding domain-containing protein
J4048_12370	2574785	2575223	-	bacilliredoxin BrxB
J4048_12375	2575401	2576298	+	YegS/Rv2252/BmrU family lipid kinase
**Cluster 11**				
**Table of genes**	**loca-tions**	**loca-tions**	**strands**	**annotations of query cluster**
J4048_15715	3202696	3203248	-	cysteine hydrolase
J4048_15720	3203344	3203743	-	YueI family protein
J4048_15725	3203808	3204057	-	YueH family protein
J4048_15730	3204129	3204351	-	spore germination protein
J4048_15735	3204412	3205519	-	AI-2E family transporter
J4048_15740	3205655	3205892	-	DUF2642 domain-containing protein
J4048_15745	3206029	3206560	-	HD domain-containing protein
J4048_15750	3206724	3207456	-	(S)-benzoin forming benzil reductase
J4048_15755	3207516	3207990	-	type VII secretion protein EssA
J4048_15760	3207997	3211114	-	type VII secretion protein EsaA
J4048_15765	3211110	3215586	-	type VII secretion protein EssC
J4048_15770	3215640	3216927	-	type VII secretion protein EssB
J4048_15775	3216941	3217181	-	EsaB/YukD family protein
J4048_15780	3217256	3217550	-	WXG100 family type VII secretion target
J4048_15785	3217865	3219095	+	PucR family transcriptional regulator
J4048_15790	3219198	3220329	+	alanine dehydrogenase
J4048_15795	3220446	3221124	+	YukJ family protein
J4048_15800	3221168	3221384	-	MbtH family protein
J4048_15805	3221402	3228530	-	amino acid adenylation domain-containing protein
J4048_15810	3228544	3229471	-	isochorismatase
J4048_15815	3229488	3231114	-	(2,3-dihydroxybenzoyl)adenylate synthase
J4048_15820	3231132	3232329	-	isochorismate synthase DhbC
J4048_15825	3232352	3233138	-	2,3-dihydro-2,3-dihydroxybenzoate dehydrogenase
J4048_15830	3233273	3234143	-	alpha/beta hydrolase
J4048_15835	3234318	3234915	-	sulfite oxidase-like oxidoreductase
J4048_15840	3235014	3235608	+	biotin transporter BioY
J4048_15845	3235654	3236983	-	TRAP transporter large permease subunit
J4048_15850	3237115	3238606	-	leucyl aminopeptidase
J4048_15855	3238753	3239230	+	divergent PAP2 family protein
J4048_15860	3239259	3239877	-	3D domain-containing protein
J4048_15865	3240001	3240322	-	YuiB family protein
J4048_15870	3240390	3240516	-	YuiA family protein
J4048_15875	3240689	3241910	-	NAD(P)/FAD-dependent oxidoreductase
J4048_15880	3242221	3243217	+	ferredoxin--NADP reductase 2
J4048_15885	3243257	3243401	-	hypothetical protein
J4048_15890	3243618	3244599	+	GMP reductase
J4048_15895	3244661	3244883	-	hypothetical protein

J4048_15900	3244899	3245427	-	stage II sporulation protein M
J4048_15905	3245423	3246128	-	ABC transporter ATP-binding protein
J4048_15910	3246120	3247776	-	hypothetical protein
J4048_15915	3247858	3248194	-	uberolysin/carnocyclin family circular bacteriocin
J4048_15920	3248261	3248834	-	hypothetical protein
J4048_15925	3249093	3249621	-	GNAT family N-acetyltransferase
J4048_15930	3249995	3250757	+	SDR family NAD(P)-dependent oxidoreductase
J4048_15935	3250900	3250975	-	iron-sulfur cluster assembly accessory protein
J4048_15940	3251019	3251385	-	hypothetical protein
J4048_15945	3251836	3252199	-	iron-sulfur cluster assembly accessory protein
J4048_15950	3252277	3253132	-	diaminopimelate epimerase
**Cluster 12**				
**Table of genes**	**loca-tions**	**loca-tions**	**strands**	**annotations of query cluster**
J4048_17470	3548731	3549091	-	phage holin family protein
J4048_17475	3549091	3549286	-	PspC domain-containing protein
J4048_17480	3549290	3550388	-	DUF4097 domain-containing protein
J4048_17485	3550412	3550739	-	hypothetical protein
J4048_17490	3550936	3551197	+	hypothetical protein
J4048_17495	3551222	3554096	-	excinuclease ABC subunit UvrA
J4048_17500	3554103	3556089	-	excinuclease ABC subunit B
J4048_17505	3556264	3556501	-	CsbA family protein
J4048_17510	3556801	3559303	+	phosphotransferase
J4048_17515	3559374	3559998	+	TetR/AcrR family transcriptional regulator
J4048_17520	3559970	3561308	+	MFS transporter
J4048_17525	3561893	3562667	-	thioesterase
J4048_17530	3562717	3563788	-	DUF3810 domain-containing protein
J4048_17535	3563873	3564935	-	saccharopine dehydrogenase NADP-binding domain-containing protein
J4048_17540	3564967	3566710	-	ABC transporter ATP-binding protein
J4048_17545	3566906	3581303	-	amino acid adenylation domain-containing protein
J4048_17550	3581314	3598429	-	amino acid adenylation domain-containing protein
J4048_17555	3598935	3599395	-	SDR family NAD(P)-dependent oxidoreductase
J4048_17560	3599467	3599629	-	hypothetical protein
J4048_17565	3600253	3600394	-	hypothetical protein
J4048_17570	3600546	3601737	-	serine protease
J4048_17575	3601811	3602165	-	Swarming motility protein SwrAA
J4048_17580	3602537	3603938	-	S41 family peptidase
J4048_17585	3604105	3605512	+	right-handed parallel beta-helix repeat-containing protein
J4048_17590	3605558	3606449	-	permease-like cell division protein FtsX
J4048_17595	3606441	3607128	-	cell division ATP-binding protein FtsE
J4048_17600	3607346	3607688	-	cytochrome c
J4048_17605	3607723	3608572	-	YitT family protein
J4048_17610	3608740	3609842	-	peptide chain release factor 2
J4048_17615	3609916	3612442	-	preprotein translocase subunit SecA
J4048_17620	3612607	3613174	-	ribosome-associated translation inhibitor RaiA
J4048_17625	3613368	3613746	-	hypothetical protein
J4048_17630	3613755	3614100	-	flagella biosynthesis regulatory protein FliT
J4048_17635	3614099	3614501	-	flagellar export chaperone FliS
J4048_17640	3614520	3616041	-	flagellar hook-associated protein 2
J4048_17645	3616291	3617446	-	flagellin

J4048_17650	3617590	3617815	-	carbon storage regulator CsrA
J4048_17655	3617808	3618240	-	flagellar assembly protein FliW
**Cluster 13**				
**Table of genes**	**loca-tions**	**loca-tions**	**strands**	**annotations of query cluster**
J4048_18730	3823751	3823940	+	4-oxalocrotonate tautomerase
J4048_18735	3823977	3824397	-	MarR family transcriptional regulator
J4048_18740	3824767	3826162	+	amino acid permease
J4048_18745	3826227	3826728	-	YwgA family protein
J4048_18750	3826767	3828069	-	HD domain-containing protein
J4048_18755	3828228	3828453	-	DUF1450 domain-containing protein
J4048_18760	3828655	3829429	+	RsfA family transcriptional regulator
J4048_18765	3829689	3830004	+	PadR family transcriptional regulator
J4048_18770	3830004	3830559	+	DUF1700 domain-containing protein
J4048_18775	3830656	3831577	+	ATP-binding cassette domain-containing protein
J4048_18780	3831573	3832527	+	ABC transporter permease
J4048_18785	3832516	3833353	-	hypothetical protein
J4048_18790	3833343	3834141	-	hypothetical protein
J4048_18795	3834109	3835033	-	hypothetical protein
J4048_18800	3835081	3835261	-	hypothetical protein
J4048_18805	3835414	3836278	-	lipoate--protein ligase family protein
J4048_18810	3836324	3837224	-	LysR family transcriptional regulator
J4048_18815	3837339	3838317	+	putative sulfate exporter family transporter
J4048_18820	3838354	3839326	-	phosphate acetyltransferase
J4048_18825	3839583	3840348	+	heme-dependent peroxidase
J4048_18830	3840467	3841247	+	SDR family oxidoreductase
J4048_18835	3841263	3842463	-	pyridoxal phosphate-dependent aminotransferase
J4048_18840	3842475	3843657	-	MFS transporter
J4048_18845	3843653	3845072	-	ATP-grasp domain-containing protein
J4048_18850	3845089	3845851	-	dihydroanticapsin 7-dehydrogenase
J4048_18855	3845847	3846558	-	cupin domain-containing protein
J4048_18860	3846547	3847162	-	bacilysin biosynthesis protein BacA
J4048_18865	3847322	3848561	-	MFS transporter
J4048_18870	3848783	3849986	+	multidrug effflux MFS transporter
J4048_18875	3850017	3851436	-	amino acid permease
J4048_18880	3851460	3853143	-	M20/M25/M40 family metallo-hydrolase
J4048_18885	3853214	3854762	-	L-glutamate gamma-semialdehyde dehydrogenase
J4048_18890	3854969	3856256	-	Glu/Leu/Phe/Val dehydrogenase
J4048_18895	3856441	3856903	-	hypothetical protein
J4048_18900	3857118	3857574	-	dTDP-4-dehydrorhamnose 3,5-epimerase family protein
J4048_18905	3857570	3858419	-	dTDP-4-dehydrorhamnose reductase
J4048_18910	3858439	3859387	-	dTDP-glucose 4,6-dehydratase
J4048_18915	3859389	3860127	-	NTP transferase domain-containing protein
J4048_18920	3860154	3861159	-	spore coat protein
J4048_18925	3861160	3861901	-	spore coat protein
J4048_18930	3861893	3863015	-	N-acetylneuraminate synthase family protein
J4048_18935	3863014	3863878	-	GNAT family N-acetyltransferase
J4048_18940	3863878	3865048	-	DegT/DnrJ/EryC1/StrS family aminotransferase
